# News and views: lysine sorbylation enters the expanding universe of posttranslational modifications

**DOI:** 10.1007/s00204-025-04187-w

**Published:** 2025-09-08

**Authors:** Ramy Ashry, Oliver H. Krämer

**Affiliations:** 1 Mainz University Medical Center, Mainz, Germany; 2 Faculty of Dentistry, Mansoura University, Mansoura, Egypt

## Abstract

Opinion Letter to Sin et al (Science Advances, 2025), Sorbate induces lysine sorbylation through noncanonical activities of class I HDACs to regulate the expression of inflammation genes.

Sorbate is a commonly used preservative that can be found in food, beverages, and cosmetics. This wide application of sorbate is justified through its minimal cytotoxicity and no evidence for carcinogenic risks. Moreover, anti-fungal activities of sorbate prevent an uptake of mycotoxins which can be carcinogenic liver toxins (Abousaty et al. 1; Bento de Carvalho et al. 2). Figure 1 shows the chemical structure of sorbate with potassium as a positively charged counter ion. Sorbate is a six-carbon, conjugated dienoate anion that is derived from sorbic acid. It has a carboxylic acid group that is also found in valproic acid (VPA) and 2-ethylhexanoic acid (EHXA). VPA is a highly useful anti-epileptic drug, a mood stabilizer, and a millimolar inhibitor of a subset of histone deacetylases (HDACs) (Göttlicher et al. 4). These epigenetic modifiers fall into four classes and their members are named according to their discovery. VPA inhibits the class I HDACs HDAC1, HDAC2, and HDAC3, and promotes proteasomal degradation of HDAC2 (Krämer 9). The aliphatic carboxylic acid EHXA occurs naturally as a racemate (mixture of two non-superimposable mirror image stereoisomers, i.e., enantiomers) and is used for industrial syntheses, paint production, and as a wood preservative (Mishra et al. 13). The R-stereoisomer of EHXA inhibits HDACs (Göttlicher et al. 4). Such enantioselective properties are also seen for derivatives of VPA (Ivanova et al. 6), which reflects the chiral nature of the catalytic domains of HDACs.

**Fig. 1:**
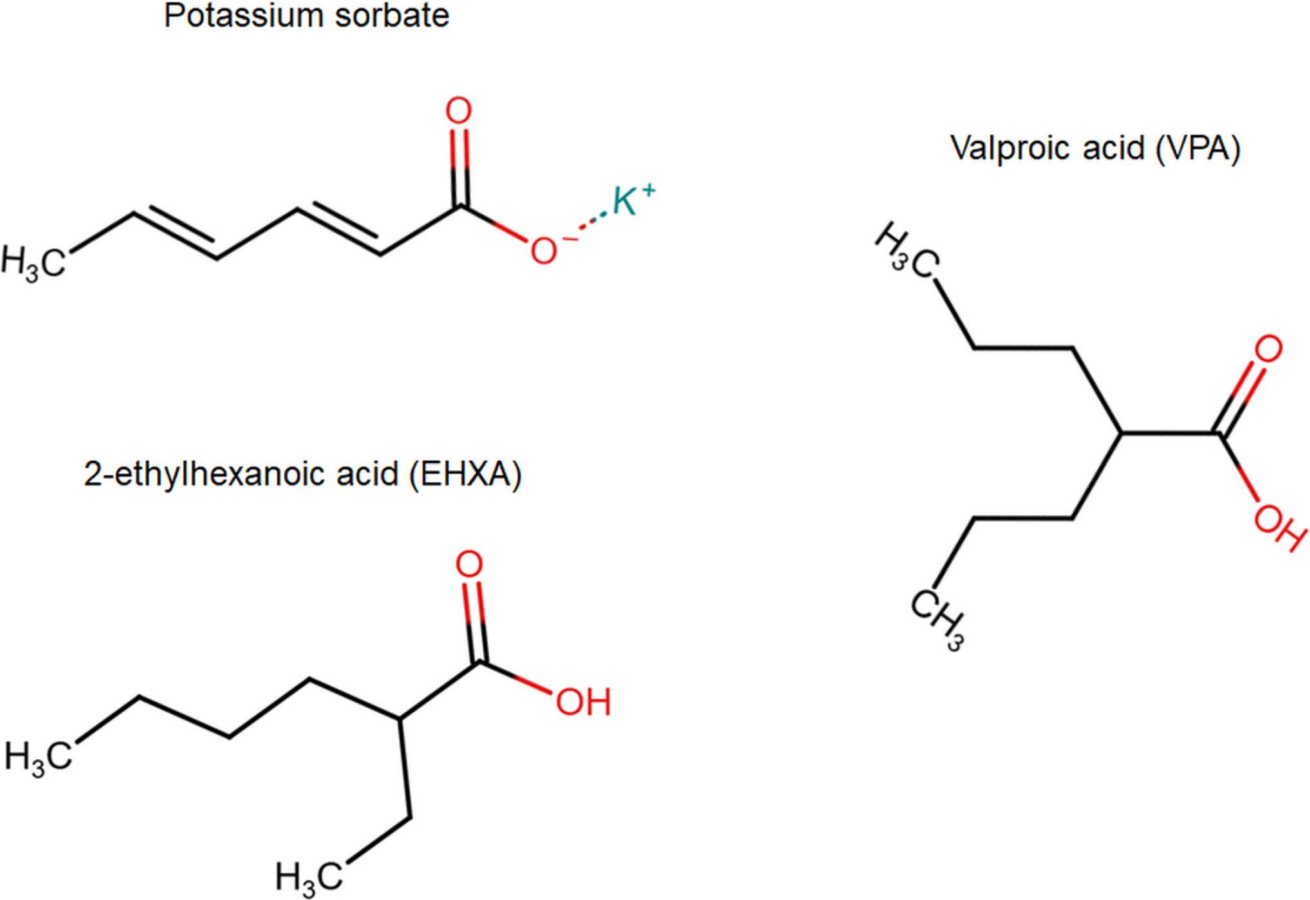
The chemical structures of potassium (K⁺) sorbate, valproic acid (VPA), and 2-ethylhexanoic acid (EHXA). The oxygen and hydrogen atoms in the carboxylic acid groups are highlighted in red. Upon deprotonation, the hydroxyl group of carboxylic acid becomes a negatively charged oxygen anion: $COOH\, \rightleftharpoons \,COO - \, + \,H\, + \,$

A study from the groups of Prof. Dr. Mashek and Prof. Dr. Chen, Minneapolis, USA, elegantly discloses that histones and non-histone proteins can undergo reversible sorbylation at their lysine residues (Sin et al. 17). Sin and colleagues show that HDACs control this posttranslational modification and reveal that the common food additive sorbate can act as an epigenetic modifier that influences physiological gene expression.

How did Sin and colleagues discover lysine sorbylation? They isolated and digested histones from sorbate-fed human colon cancer cells and used unbiased mass spectrometry. This approach revealed that sorbate was covalently attached to lysine residues within histones. This histone modification became detectable by immunoblot using an anti-sorbylated lysine antibody that Sin and colleagues developed. As testbeds, they used human embryonic kidney-derived, human liver cancer-derived, and murine macrophage-like cells that were incubated with 2–5 mM sorbate. Lysine sorbylation of histones was also detectable with lysates from the livers of mice that had received 0.5% potassium sorbate per oral gavage. The dynamic regulation of lysine sorbylation was proven by sorbate administration, as well as its withdrawal and an ensuing loss of sorbylation (Sin et al. 17). Sorbylated N-terminal lysine moieties overlap with known histone acetylation and methylation sites (Suraweera et al. 18). Therefore, it appeared plausible that sorbylation competed with such modifications. However, sorbylation of histones occurred at comparably low levels and did not alter global histone acetylation significantly (Sin et al. 17).

HDACs hydrolytically cleave acetylation marks as well as comparably larger acylation marks on proteins (Wang et al. 20). These have a similar length and structure as the sorbylation mark. Sin and colleagues hypothesized that HDACs controlled lysine sorbylation. They added sorbate to human embryonic kidney-derived cells overnight, removed it, and applied HDAC inhibitors with variable specificities for 5 h to determine if these drugs altered lysine desorbylation kinetics (Sin et al. 17). Butyrate and trichostatin-A, which inhibit most zinc-dependent HDACs (Mustafa and Krämer 14), prevented the decrease of lysine sorbylation upon sorbate washout (Sin et al. 17). The benzamide HDACi entinostat and the benzoylhydrazide HDACi UF010 selectively inhibit HDAC1, HDAC2, and HDAC3 at micromolar concentrations (Mustafa and Krämer 14; Wang et al. 19). Both agents attenuated lysine desorbylation upon removal of sorbate. Nonetheless, very high doses of up to 10 mM entinostat dose-dependently decreased sorbylation levels when applied with sorbate. This was linked to an inverse relationship between lysine sorbylation and acetylation levels (Sin et al. 17). Thus, HDAC activity seems to be necessary for dynamic regulation of sorbylation (Fig. 2). Another interpretation is that the consumption of metabolites upon hyperacetylation limits the processivity of sorbylation. We hypothesized such a mechanism of competing substrates for hyperacetylation, based on data that were collected with HDAC inhibitors of different specificities. We disclosed that hyperacetylation of the highly abundant acetylation substrate tubulin in cells with inhibited or deleted cytosolic HDAC6 tied in with reduced histone acetylation (Jungwirth et al. 7; Krämer et al. 11). Furthermore, it cannot be excluded that that addition of high concentrations of HDACi alters the structures and thereby the functions of HDACs.

**Fig. 2:**
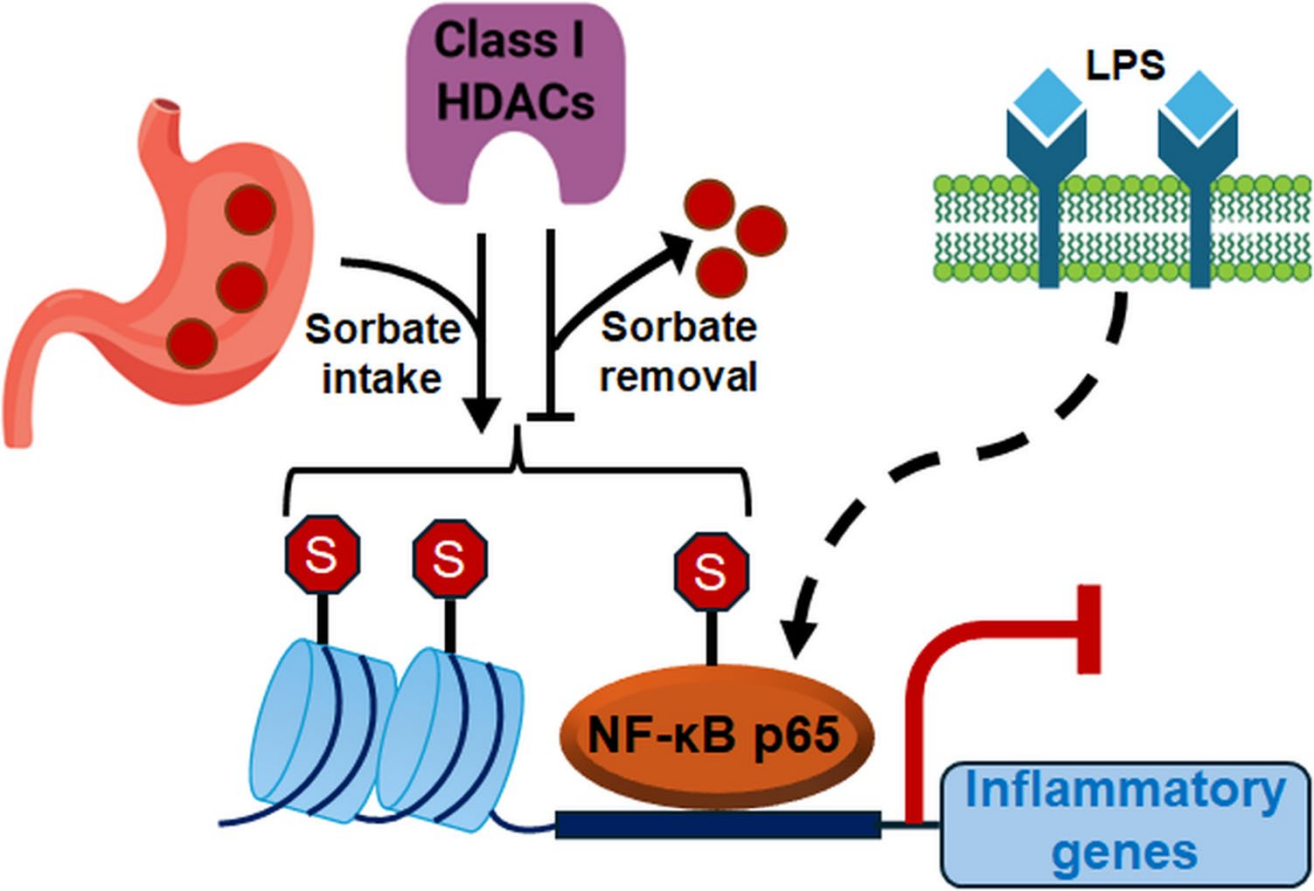
Lysine sorbylation requires the uptake of sorbate into cells after absorption from the gastrointestinal compartment. Depending on the availability of sorbate, the class I HDACs HDAC1, HDAC2, and HDAC3 promote or remove sorbylation from lysine moieties in proteins. These include histones and the inducible transcription factor NF-κB p65. This newly recognized molecular mechanism attenuates lipopolysaccharide-induced, NF-κB-dependent gene expression; S, sorbate/lysine sorbylation; NF-κB, nuclear factor kappa B; LPS, lipopolysaccharide

A knockdown of HDAC1, HDAC2, and HDAC3 by RNAi, the isolation of HDAC1, HDAC2, and HDAC3 by immunoprecipitation, and subsequent assessment of lysine desorbylation in enzymatic assays verified that these enzymes promoted lysine sorbylation in vitro in the presence of 10–20 mM sorbate. HDAC2 appeared to be the strongest regulator of lysine sorbylation in vitro (Sin et al. 17). Unfortunately, single knockdowns of these HDACs and single HDAC null cells were not appreciated in the study. A more thorough understanding of this process might have been achieved with the use of such cell systems and catalytically dead HDAC mutants (Hess et al. 5; Kiweler et al. 8). Irrespective of such limitations, Sin and colleagues made the surprising discovery that HDACs can exhibit a noncanonical dual functionality as desorbylases (in absence of sorbate) and as sorbyltransferases (in presence of sorbate). Experiments with HDAC inhibitors blocking the catalytic domains of HDACs support the idea that this structural feature of HDACs determines lysine sorbylation levels.

What could be the physiological relevance of lysine sorbylation? Unbiased RNA-sequencing analyses of livers from mice which had received 0.5% sorbate for 4 h revealed a downregulation of inflammation-related gene expression patterns. Sorbate also decreased the induction of proinflammatory genes and nitric oxide production in murine macrophages that were exposed to the lipopolysaccharide of Gram-negative bacteria (Sin et al. 17). This activator of the mammalian immune system can help to eliminate pathogens but occasionally contributes to excessive immune reactions (Pfalzgraff and Weindl 16). Systematic analysis of proteins carrying lysine sorbylation, by immunoprecipitation followed by mass spectrometry, revealed over 1600 sites on 908 proteins. These include the NF-κB transcription factor p65. The sorbylated NF-κB interaction cluster comprises p65, the transcription regulators BRD4 and SMRT, the inducible transcription factor STAT1, and the DNA damage sensor ATM. Cellular assays illustrated sorbylation of p65 at the lysine residues 310, 314, and 315 (Sin et al. 17). NF-κB members and STAT1 are decisive nodes for beneficial and detrimental immune reactions (Pfalzgraff and Weindl 16). Assays with a synthetic luciferase construct showed that sorbate attenuated the lipopolysaccharide-evoked transcriptional activation of NF-κB (Fig. 2). To firmly demonstrate that sorbylation modulates NF-κB, endogenous target genes of sorbylated p65 and NF-κB p65 with mutations in lysine residues 310/314/315 must be analyzed. Because NF-κB has tissue-specific roles and due to sorbate’s ingestion route with broad systemic distribution, inflammatory pathways in e.g., immune, brain, or gut tissues should be analyzed in chronic in vivo exposure scenarios. Such studies can overcome the current limitation to acute liver responses and a macrophage-type cell line. Further research is likewise needed to define why p65, and not the other NF-κB family members, undergoes lysine sorbylation. The structure of NF-κB p65, but also its association with I-κB or its acetylation-regulated interaction with STAT1 are potential explanations (Chen et al. 3; Krämer et al. 10; Kumar et al. 12; Noack et al. 15). Furthermore, sorbylation may compete or cross-talk with other posttranslational modifications and there might be reader proteins for sorbylated lysine residues. The conjugated double bond of sorbate with its delocalized electron-rich π-π system may adjust the formation of higher-order complexes on chromatin.

In sum, the study by Sin and colleagues reveals lysine sorbylation as dynamic and reversible modification that is controlled by the noncanonical dual activity of class I HDACs as desorbylases or sorbyltransferases (Fig. 2). The widespread use of sorbate raises key questions about its long-term impact on health through epigenetic mechanisms. Both public health consideration and therapeutic exploration are warranted. Longer-term studies assessing chronic sorbate exposure through dietary intake, assessing the minimal effective concentration required for biological effects, and the pharmacokinetic dynamics of sorbylation can decipher implications of lysine sorbylation in inflammation and age-related diseases.

## Data Availability

A data availability statement is not applicable.
